# Mechanisms of an inhibitory control training to reduce binge eating behaviours: study protocol of the randomized controlled proof-of-principle MIND BINGES trial

**DOI:** 10.1186/s40337-025-01358-z

**Published:** 2025-08-07

**Authors:** Sebastian M. Max, Katrin E. Giel, Christian Plewnia, Simone Weller, Andreas J. Fallgatter, Veronika Lossa, Stephan Zipfel, Kathrin Schag

**Affiliations:** 1 Department of Psychosomatic Medicine and Psychotherapy, University Hospital Tübingen, Osianderstraße 5, 72076 Tübingen, Germany; 2 Centre of Excellence for Eating Disorders Tübingen (KOMET), Tübingen, Germany; 3 German Center for Mental Health (Deutsches Zentrum Für Psychische Gesundheit, DZPG), Partner Site Tübingen, Germany; 4 Department of Psychiatry and Psychotherapy, Neurophysiology and Interventional Neuropsychiatry, Tübingen Center for Mental Health, University Hospital Tübingen, Calwerstraße 14, Tübingen, Germany; 5 Lived Experience Representative, Tübingen, Germany

**Keywords:** Bulimia Nervosa, Binge eating disorder, Eating disorder, Cognitive control, RCT, Treatment, Cognitive training, Feedback

## Abstract

Current guidelines recommend psychotherapy as first-line treatment for eating disorders. Many individuals with eating disorders characterized by binge eating episodes benefit from psychotherapy. However, a substantial subgroup does not achieve abstinence from binge eating. Bulimia Nervosa (BN) and Binge eating disorder (BED), along with other Specified/Unspecified Feeding or Eating Disorders (OSFED/UFED) with regular binge eating behaviour can be clustered on a continuum of eating disorders which is characterized by elevated impulsivity. Elevated impulsivity, and especially decreased inhibitory control, seem to be key factors in developing and maintaining binge eating behaviour. To target and modify these proposed underlying altered processes of binge eating, cognitive training approaches promise to achieve an amelioration of eating disorder-related psychopathology. Feedback is thought to play a crucial role in cognitive training approaches to increase learning and maintain motivation but its exact role has not been examined yet. MIND BINGES is a prospective, randomized controlled proof-of-principle trial investigating a food-modified inhibitory control training (ICT) and in particular the role of feedback on each individual’s performance. 63 patients with regular binge eating (BN, BED, OSFED, UFED) will be randomly assigned to receive either ICT with feedback after each session, ICT without feedback, or be assigned to a waitlist-control group (WL). The ICT is based on an antisaccade paradigm consisting of six training sessions over the course of two weeks. As the role of individual feedback provided to patients is unclear, the core aims are to investigate: (1) whether ICT without feedback is non-inferior to ICT with feedback in reducing binge eating episodes, and (2) whether ICT without feedback is superior to WL in reducing binge eating episodes. Thus, the primary endpoint is binge eating frequency in terms of changes in binge eating episodes four and 12 weeks after ICT/WL termination compared to baseline. Secondary outcomes include eating disorder psychopathology, general psychopathology, inhibitory control capacities, impulsivity, quality of life, self-esteem, and self-efficacy. Lastly, regression analyses will be conducted to explore the impact of secondary outcomes on the likelihood of a positive treatment effect. The results of the present trial will help to disentangle different elements of ICT, in particular the role of feedback. Based on the secondary outcomes, this trial might contribute to a better understanding of pathways and underlying mechanisms of an ICT. In sum, this helps developing possible individually tailored and effective treatment approaches for individuals suffering from regular binge eating. Findings might also inform cognitive trainings targeting symptoms of other mental health disorders.

**Supplementary Information:**

The online version contains supplementary material available at 10.1186/s40337-025-01358-z.

## Abstract (plain language summary)

Psychotherapy is the current first choice in treatment for eating disorders. Many people experiencing binge eating, which comprises eating a large amount of food in a short time and the feeling of loss of control over the eating, benefit from psychotherapy. Yet, that’s not the case for all of them. There are several eating disorders that are characterized by binge eating, for example Bulimia Nervosa (BN), and Binge Eating Disorder (BED). Those eating disorders are characterized by an increased impulsivity which might lead to those binge eating behaviours. One way to increase regulation of impulsivity, so called “inhibitory control training “ (ICT) might be helpful, which comprises a training programme on the computer with eye-tracking. The MIND BINGES study is a research trial to investigate this food-related ICT with 63 patients that regularly binge eat. They will be randomly assigned to one of three groups. Either ICT with feedback about their task performance after each session or ICT without feedback or a control group that does not receive the ICT. The ICT consists of six sessions where the patients train to look away from food pictures over the course of two weeks. The primary goal is to compare, if the training without feedback is as effective as the training with feedback. Further, we want to prove the effectiveness of the training compared to the control group. Therefore, we investigate how much decrease in binge eating frequency will be observed in each group four or twelve weeks after finishing the training programme. Additionally, it will be also looked at other factors like mental health, self-control, impulsivity, quality of life, self-esteem and self-efficiency. With this study, we hope to get a better understanding on how ICT works and whether feedback makes a difference. This could lead to better and more personalized treatments for people suffering from binge eating and possibly other mental health issues.

**Supplementary Information:**

The online version contains supplementary material available at 10.1186/s40337-025-01358-z.

## Introduction

Impaired inhibitory control and disinhibited eating is predominantly seen in a subgroup of individuals with overweight and obesity suffering from regular binge eating behaviours. Binge eating according to DSM-5 is defined as eating a large amount of food accompanied by a subjective loss of control [4]. Transdiagnostic approaches suggest that individuals with regular binge eating, regardless of specific eating disorder diagnoses, represent a shared clinical entity. Regular binge eating is the primary symptom in Binge Eating Disorder (BED), Bulimia Nervosa (BN), and in Other Specified or Unspecified Feeding and Eating Disorders (OSFED/UFED) with regular binge eating behaviour [40, 42, 54]. Impairments in inhibitory control are considered a vulnerability factor linked to disinhibited eating patterns and, as a consequence, weight gain and difficulties in weight regulation [21]. These vulnerabilities highlight important targets for tailored interventions and treatment strategies.

### The role of inhibitory control in binge eating

Inhibitory control refers to the ability to suppress dominant reaction in favour of another reaction [39]. It enables individuals to act on intentions that conflict with habits, motivations, or competing goals [22]. One important process to achieve inhibitory control consists in top-down focusing on relevant stimuli while ignoring irrelevant stimuli [36]. Deficits in inhibitory control are considered a vulnerability factor for obesity and binge eating [12, 18, 21, 43]. If food stimuli are perceived as highly rewarding, they attract a strong attentional focus and if inhibition mechanisms fail, overeating may occur [15, 20].

### Underlying neurobiological markers and their implementation in inhibitory control trainings

The prefrontal cortex and especially the dorsolateral prefrontal cortex is closely linked to inhibitory control processes [14] and plays a key role in processing food stimuli [17]. Current evidence highlights the dorsolateral prefrontal cortex’ central role in the regulating eating behaviour, and especially in inhibitory control [20, 28, 29, 32]. Its activation during both short- and long-term attempts to resist food intake makes it a promising target for modulating inhibitory control over eating. One approach is inhibitory control training (ICT) [43], such as the antisaccade paradigm, where participants are instructed to avert their gaze as soon as a stimulus is shown, therefore demanding a suppression of an undesirable but highly dominant behaviour and initiating and executing an alternative behaviour [27]. This training approach recently proved to be feasible and acceptable in a clinical eating disorder population, improving inhibitory control towards high-caloric food images and a reducing binge eating frequency [19]. These effects were enhanced by application of transcranial direct current stimulation (tDCS) of the right dorsolateral prefrontal cortex, supporting the central neuropsychological involvement of neural circuits in inhibitory control [19, 38].

### The role of feedback in cognitive trainings

Feedback regarding task performance in the food-modified antisaccade training might be a crucial component to learn the respective task, to enhance or maintain motivation and finally, to reduce binge eating frequency. „Feedback is useful information that helps individuals recognize errors, on how they possibly can improve their performance or deepen their understanding in a specific task [51]. It is considered central to learning in perceptual-cognitive training programmes and may improve compliance in individuals with ADHD [35]. A recent meta-analysis highlights feedback as a key element of gamification in cognitive trainings to achieve behavioural change and intrinsic motivation [52]. However, other forms of gamification may further enhance outcomes. A broad meta-analysis in educational research including over 400 studies concludes that feedback has high impact on skills (i.e. the performance in a cognitive task), but only low effects on motivational or behavioural outcomes [55]. Similarly, in our previous trial, performance in the antisaccade training did not correlate with reductions in binge eating frequency, suggesting that task-related feedback might not be necessary to reduce binge eating frequency [19]. In our training, feedback consisted of a session-based error percentage after each training session. This, the feedback was simple and could have been positive or negative depending on the performance and in relation to the performance in the session before. Additionally, even though evidence is inconsistent regarding immediate vs. delayed feedback [55], intermittent feedback (e.g. after task completion) might be more effective compared to regular feedback (e.g. after each trial) [35]. Feedback used in the food-modified antisaccade training is delayed but regular (i.e. not presented after each trial but rather after each training session). Interestingly, in a subclinical sample consisting of restrained eaters, the feedback in the inhibitory control training was more useful when participants received no feedback compared to immediate feedback after each trial [1]. Taken together, evidence regarding the role of feedback in cognitive trainings is controversial and the feedback in our training might not contribute to its efficacy and might therefore not be a necessary component. The data in our previous trial however does not enable us to draw conclusions on feedback as a mechanism of change in the food-related antisaccade training [19].

### Study aims

Multiple aspects should be addressed to advance our understanding of eating disorders. First, existing treatments for individuals affected by binge eating often lack both effectiveness as well as long-term impact, highlighting the need for novel therapeutic approaches. The current trial targets underlying neurobiological mechanisms, particularly inhibitory control, which appears to be a key factor in the development and maintenance of binge eating behavior. This is directly addressed through ICT. Moreover, we examine the role of feedback, a critical component in modulating motivation and its influence on treatment outcomes. In particular, we investigate non-inferiority of ICT without feedback compared to CT with feedback; inferiority of ICT compared to WL. Finally, given that the mechanisms behind effective treatment are still not fully understood, we aim to identify predictors and moderators of treatment success. This will support the development of individualized treatment strategies—not only by deepening our understanding of the core mechanisms that contribute to treatment response or even remission, but also by informing the implementation of these strategies into scalable, app-based self-guided interventions.

### Current study

As the food-modified antisaccade training alone produced strong effects in our previous trial [19], including decreases of binge eating frequency, eating disorder pathology, and antisaccade performance independently from the tDCS condition, we now investigate mechanisms underlying the effects of the food-modified antisaccade training without neuromodulatory support through tDCS. This proof-of-principle trial will investigate the role of feedback in the food-modified antisaccade training to reduce binge eating. In a randomized controlled parallel group design two conditions of the inhibitory control training are implemented: a) with feedback and b) without feedback. Both will be compared to c) no treatment. The superordinate research question of the project is to investigate the role of feedback in the food-modified antisaccade training to reduce regular binge eating behaviours. In more detail, we propose:

- Noninferiority of the food-related antisaccade training without feedback compared with the original training including feedback in patients with regular binge eating regarding binge eating frequency (primary outcome) and eating disorder pathology, impulsivity, task performance, motivation and self-efficacy (secondary outcomes).
- Confirmation of clinically relevant effects of the training without feedback against the waitlist control and confirmation of clinically relevant effects of the training with feedback against the waitlist control i.e. superiority in comparison with a group with no treatment regarding primary and secondary outcomes.
- We will perform regression analyses concerning impact of sociodemographic variables and psychopathology and the treatment outcome depending on the training condition.
- Subordinate, we are interested in mechanism-driven pathways of effective treatment with ICT in patients with regular binge eating behaviour. Thus, we exploratory investigate the pathways between the primary outcome and secondary outcomes, that also serve as predictors and moderators.

## Methods/design

The present study protocol is reported according to the SPIRIT checklist [13].

### Study design and setting

The MIND BINGES trial is a single centre clinical proof-of-principle double-blind RCT with three parallel arms.

Participants are recruited from the outpatient eating disorder service of the Medical University Hospital Tübingen, via study announcements in the media, via email lists of the University Tübingen, and individuals who have previously participated in eating disorder research studies at the Department of Psychosomatic Medicine and Psychotherapy and gave written consent to be informed about future studies. Interested participants receive verbal and written information material and are screened for inclusion and exclusion criteria through a standardized checklist. After providing written informed consent, the baseline assessment takes place. After completion of the baseline assessment, patients will be randomized in a 1:1:1 ratio to one of the three conditions. Outcomes will be measured at baseline (T0), at each training session (T1-T6), at end of treatment (T7) and at two follow-up timepoints, a four-week follow-up (T8) and a 3-months follow-up (T9).

### Study participants and eligibility criteria

The study population consists of individuals who report regular binge eating behaviour (at least 2 objective binge eating episodes per month within the past three months) and fulfil a diagnosis of BED, BN, OSFED or UFED according to the DSM-5 [4].

The eating disorder will be diagnosed by the *Eating Disorder Examination (EDE*; see 3.7.) [26], a standardized structured expert interview. OSFED/UFED is diagnosed, if one of the following cases occurs:

- The amount of objective binge eating episodes is below once per week during the last three months
- Less than three B-Criteria of binge-eating-episodes are fulfilled (Eating much more rapidly than normal; eating until feeling uncomfortably full; eating large amounts of food when not feeling physically hungry; eating alone because of feeling embarrassed by how much one is eating; feeling disgusted with oneself, depressed or very guilty afterwards)
- In case of potential BN: the amount of binge eating or purging is below once per week during the last three months or the individual does not suffer from body dysmorphia/dissatisfaction.

#### Inclusion criteria

Patient eligible for the trial must comply with all of the following at randomization:

- Age ≥ 18 years
- BMI > 18 kg/m.^2^
- Written informed consent

#### Exclusion criteria

- Insufficient knowledge of German or English language
- Current pregnancy or lactation period
- Current or lifetime psychotic disorder, bipolar-I disorder, current substance dependence, current suicidality
- Previous bariatric surgery
- Severe somatic disorders (e.g. insulin resistance) which influence weight or eating behaviour and are not controlled by stable medication
- Severe neurologic diseases
- psychoactive medication that might interfere with cognitive functioning, e.g. neuroleptics, benzodiazepines, opioids as well as medication that might influence hunger/weight, e.g. GLP-1 receptor agonists
- Uncorrectable or impaired vision, ametropia, eye diseases which prevent from executing the task

### Interventions

Eligible patients who are randomized to one of the two training conditions receive six sessions of a food-related inhibitory control training within a two-weeks span and they will be randomly assigned to receive either feedback about their task performance in the food-related antisaccade task after each training session or receive no feedback. Patients being randomized to the control group will not receive any intervention within the two-weeks span.

#### Psychoeducation

A short psychoeducation (approximately 5–10 min) is provided by a trained psychologist to patients in the training condition with or without feedback. It consists of an overview on eating disorders that entail binge eating behaviour, as well as mechanisms of the inhibitory control training and the transfer of training effects to everyday life.

#### Inhibitory control training

**Fig. 1:**
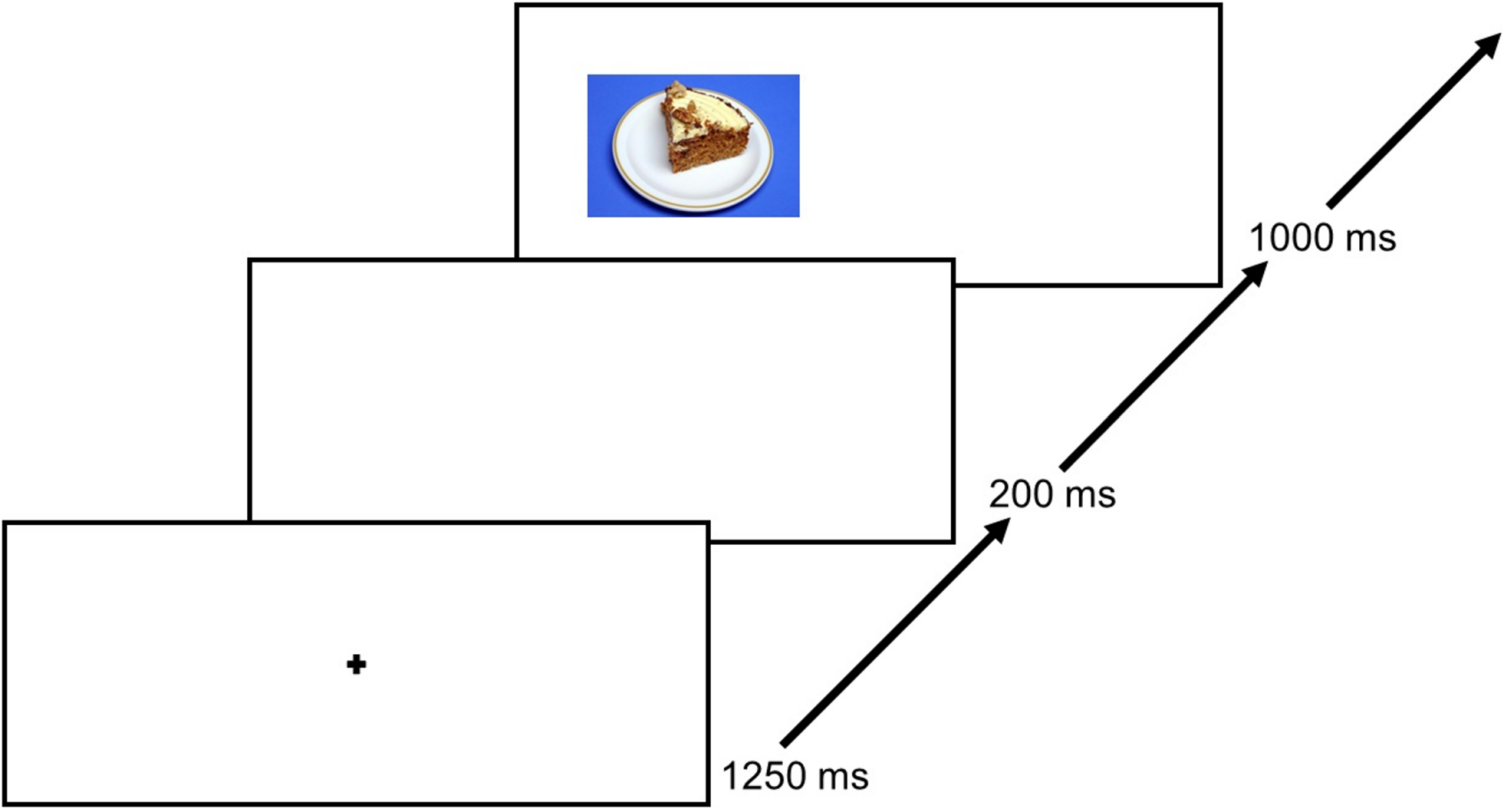
Exemplary trial course of the food-modified antisaccade paradigm. Previously used by Max et al. [38]

The inhibitory control training is based on the principles of the antisaccade paradigm, an established experimental computer-based eye tracking task which is composed of two main components [5]: First, each trial starts with a central fixation cross which is thereafter succeeded by a stimulus presented slightly left or right from the cross in a peripheral screen position (see Fig. 1). If a novel stimulus appears in the peripheral visual field, as it is the case within the antisaccade paradigm, the reflexive reaction is to direct the gaze towards this stimulus, ergo, performing a prosaccade. The antisaccade paradigm demands to suppress this highly dominant behaviour which requires adequate inhibitory control. Previous studies have already shown that antisaccade performance is highly correlated with performance in other established tasks that assessed inhibitory control. In particular, brain networks involved in volitional behavioural and inhibitory control are activated during the antisaccade task [2, 27].

In the food-modified antisaccade paradigm employed in a previous trial, we found that obese participants with BED demonstrated a higher prevalence of antisaccade difficulties. Additionally, participants exhibited greater challenges in averting their gaze from food images they had wrongfully looked at, supporting the assumption that binge eating behaviour is characterized by major impairments in inhibitory control and that this impairment might maintain maladaptive eating behaviours [50].

Based on this evidence, we previously conducted a trial with the food-specific inhibitory control training coupled with transcranial direct current stimulation (tDCS) [19], which showed promising results regarding improvement of inhibitory control as well as the amelioration of the eating disorder related pathology.

During the training session, patients with binge eating behaviour practice the suppression of a prepotent oculomotor response towards a peripheral food stimulus and with that we aim to reduce error rates, that is, the reduction of unwanted prosaccades towards food stimuli.

Analogue to Giel et al. [19], patients rate 40 colour pictures depicting high-caloric food concerning valence, appetite, liking, and wanting of the food item. Subsequently, the 20 highest-rated stimuli are individually chosen for the training period. During training, each picture is presented four times within one block, while presentation location on the screen (left/right) is counterbalanced. We present the stimuli in four blocks, resulting in 320 trials. Each trial starts with a central fixation cross (1250 ms), succeeded by a 200 ms interstimulus interval. Afterwards, the food picture is presented for 1000 ms. One group will receive feedback about their error rates after each training session, whereas the other group will not receive any feedback.

During task performance, the participant’s gaze is recorded using a iViewX Hi-Speed eye tracking system (SensoMotoric Instruments, 2010) with a sampling rate of 500 Hz and 0.25°–0.5° gaze position accuracy.

Hunger and mood are assessed at the beginning of every training session using visual analogue scales.

### Patient safety

Eye tracking is a non-invasive method used to record eye movements. We employ an infrared-assisted, video-based system in which a low-intensity infrared light is directed at one eye via a transparent mirror, producing a reflection point on the cornea. The device is CE-certified and used in accordance with its intended purpose. No safety risks are anticipated.

Regarding the interviews and questionnaires used, it is highly unlikely that they will cause any harm. However, in the event of unexpected emotional distress, the study will be discontinued immediately.

Potential (severe) adverse events will be documented by the experimenter as soon as reported during the study assessments or training sessions. The adverse event form contains following information [41]: detailed description of the event and affected patient,start and stop date and time; severity (yes/no); intensity (mild/moderate/severe); actions taken to manage the adverse event (yes/no); result of actions taken (fully healed/in the healing process/not healed/permanent damage/lethal/unknown); causality (given/probable/possible/unlikely/not given/not estimable). Study patients were encouraged to contact study staff in case of worsening of their condition at any time during the study.

### Concomitant care

Participants are allowed to receive other parallel psychotherapeutic treatments for their eating disorder (inpatient, outpatient, self-guided). Other behavioural or supportive care is also allowed, e.g. consultations of general practitioners, nutritional advice, physical activity or dieting programmes. Type of parallel treatment is documented. Concurrent use of psychoactive medications is allowed during the trial with the exception of neuroleptics, benzodiazepines, and opioids as they might interfere with cognitive functioning. Type and dosage of medication is assessed at baseline. If participants with concomitant care are not equally distributed across groups, we will compute sensitivity analyses, e.g. we will rerun our analyses after exclusion of those patients receiving concomitant care, or we will compare the results of those receiving no concomitant care with those receiving concomitant care.

### Participant timeline

The participant timeline is depicted in Fig. 1.

Study duration for each patient comprises 14 weeks. After a baseline assessment (T0, 2 h) including a diagnostic assessment a randomization to either of the three study arms is conducted. Patients get randomized into a) 6 sessions of the food-modified antisaccade training (T1–T6, 20 min each) with feedback, b) 6 sessions of the food- modified antisaccade training (T1-T6, 20 min each) without feedback or c) without treatment during the two weeks. After this two-week span, a post-intervention assessment (T7, 75 min) will be conducted and thereafter a second post-intervention assessment four weeks after training termination (T8, 45 min) and a three-months follow-up assessment focusing on changes in eating disorder symptomatology via telephone (T9, 10 min) will be conducted. In total, the duration of the assessments for the ICT-conditions is around six hours and 10 min across nine appointments. For the waitlist control group, the four appointments take around 4 h and 10 min in total. Details on the investigated outcomes of each time point are presented at Table 1.

**Table 1:** Schedule of enrolment, interventions, and assessments within the MIND Binges trial according to SPIRIT

		Baseline	Allocation	Intervention Period	Post-intervention I	Post-intervention II	12-week Follow-Up
**TIMEPOINT**		**T0**		**T1-T6**	**T7**	**T8**	**T9**
ENROLMENT:							
Eligibility screen		X					
Informed consent		X					
Randomization			X				
INTERVENTIONS:							
Inhibitory control training with Feedback							
Inhibitory control training without Feedback							
Waitlist control							
ASSESSMENTS (Instrument):							
Primary outcome:							
BE frequency in the last 4 weeks (EDE)		X				X	X
Secondary outcomes:							
Eating disorder pathology	Eating disorder pathology (EDE)	X				X	
	binge eating frequency in the last 7 days (Process analyses of impulsive behaviours)	X		X (only once per week)	X	X	
	Proportion of (partial) remission of BED (EDE)					X	X
General psycho-pathology	mental comorbidities (SCID-5-CV)	X					
	Depressive symptoms (BDI-II)	X			x	X	
Eating behaviour	Impulsive eating (TFEQ)	X			X	X	
Body Mass Index		X				X	X
Gender (GERAS)		x					
Quality of life (WHO-5)		X			X	X	
Stimulus valence ratings		x			x		
impulsivity	Inhibitory control (Antisaccade task performance)	X		X	X		
	Impulsive behaviours (Process analyses of impulsive behaviours)	X		X (only once per week)	X	x	
	negative urgency (UPPS)	X			x	X	
Motivation (URICA-S)		x			x	x	
Self-Esteem (RSE)		x			x	x	
Treatment expectancy (Self-developed questionnaire)		x					
Self.efficacy	General self-efficacy (IE-4)	X			X	x	
	Food-related efficacy (Self-developed questionnaire)	X			X	x	
Treatment evaluation (Self-developed questionnaire)					x		

*BDI-II *Becks’s Depression Inventory, Version 2; *EDE* Eating Disorder Examination, *GERAS* Gender-Related Attributes, *IE-4* Internal–External locus of control, *RSE* Rosenberg Self-Esteem Scale, *TFEQ* Three-factor eating questionnaire, *UPPS* UPPS impulsive behavior scale, *URICA-S* University of Rhode Island Change Assessment, *WHO-5* WHO (Five)-Well-Being Questionnaire

### Outcomes

**Fig. 2:**
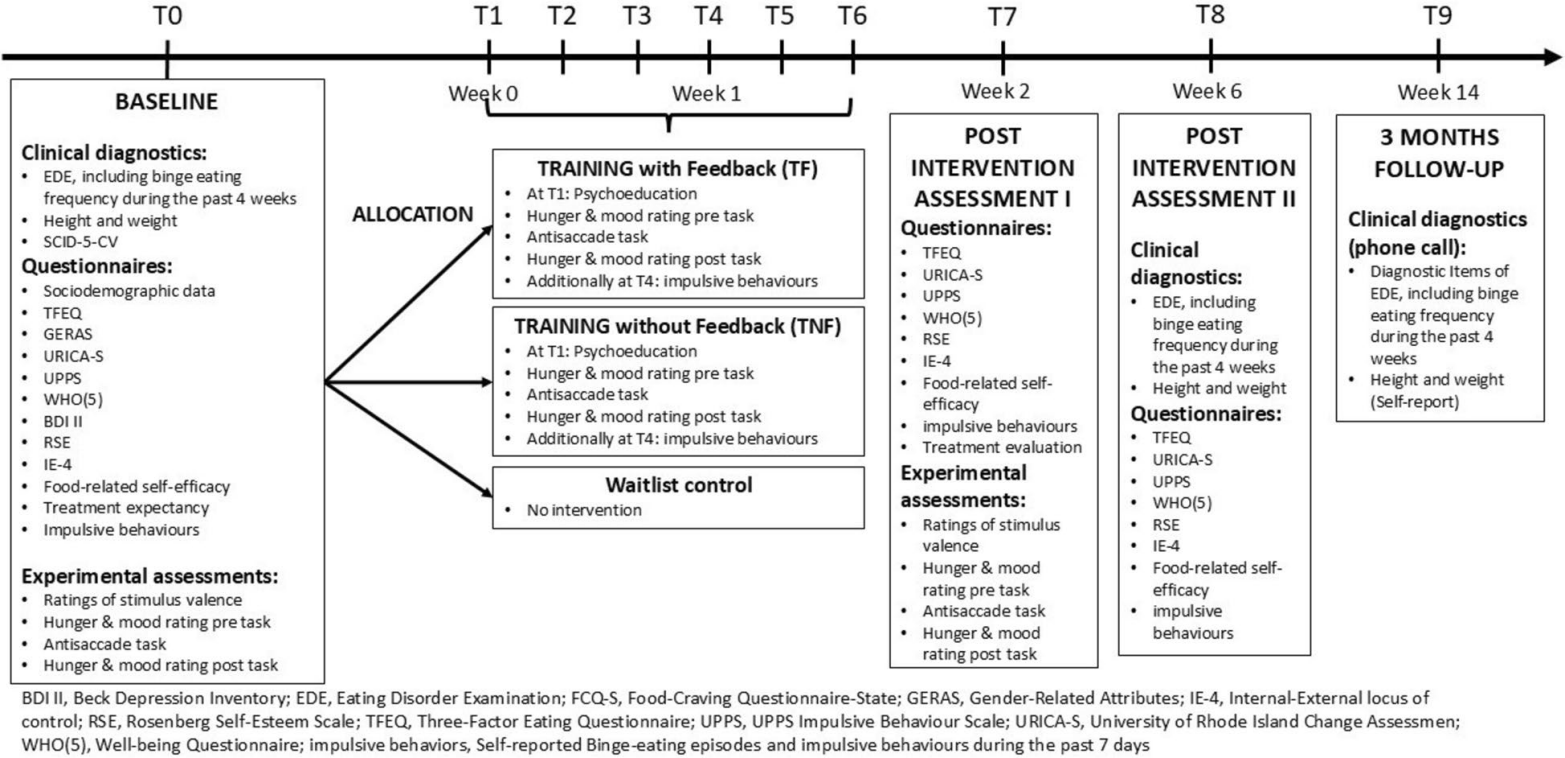
Participant timeline of the MIND BINGES trial

Table 1 and Fig. 2 give an overview on assessment points and outcome measures.

#### Primary outcome measure

Binge eating frequency in the last four weeks according to the Eating Disorder Examination (EDE) [26] will serve as a primary clinical outcome. EDE is a standardized structured expert interview to assess eating disorder psychopathology. It has shown high inter-rater reliability (Cohens Kappa > 0.80) and internal consistency(Cronbach’s Alpha 0.73 for the subscales; 0.93 for the total score) indicating a good quality of the interview on a superordinate level [26]. EDE includes diagnostic items as well as items related to the four subscales restraint, eating concern, weight concern and shape concern. The diagnostic item 10 is assessing the binge eating frequency in the past four weeks. We compare changes in binge eating episodes at the four weeks follow-up (T8) to baseline (T0) and at the 3-months follow up (T9) to baseline (T0). Binge eating frequency is of high clinical relevance as binge eating resembles the core pathology of eating disorders with regular binge eating [4].

#### Secondary outcome measures

Secondary outcomes will be assessed by validated structured clinical interviews, validated self-report instruments as well as established neuropsychological tasks.

##### Eating disorder pathology

The eating disorder diagnoses will be assessed by the EDE (see primary outcome) [26], which is applied by trained raters. Further, a self-developed protocol assessing binge eating frequency in the last seven days concerning short-term changes will be used [48].The following measures will be assessed additionally to the primary outcome:

- Eating disorder diagnoses at T0, T8 and T9
- Severity of eating disorder pathology (general eating disorder pathology, restraint, eating concern, shape concern, weight concern) at T0 and T8.

##### General psychopathology

Comorbid mental disorders are assessed by the *SCID-5-CV* [7]. The *Beck’s Depression Inventory*, Version 2 (BDI-II), is used to assess depressive symptoms [24]. Following measures will be assessed:

- Number of current and lifetime comorbid mental disorders at T0
- Severity of depressive symptoms at T0, T7 and T8

##### Gender

Gender will be assessed by the *Gender-Related Attributes* (GERAS) [23]. Following measures will be assessed:

- Gender-related personality traits (Personality traits, cognitive abilities, interests) at T0

##### Eating behaviour

Eating behaviour will be assessed using the *Three-Factor Eating Questionnaire* (TFEQ) [45]. Following measures will be assessed:

- Quantification of cognitive restraint of eating, disinhibition and hunger at T0, T7 and T8

##### Body mass index

*Body Mass Index* (BMI) will be obtained by calculating objectively assessed body weight and height at T0 and T8, and self-reported at T9.

##### Quality of life

Quality of life is assessed by the *WHO (Five)-Well-being Questionnaire* (WHO-5) [11]. Following measure will be assessed:

- Quantification of general mental-well being at T0, T7 and T8

##### Impulsivity and inhibitory control

Impulsivity and inhibitory control will be assessed based on different approaches: On a behavioural level, performance measure of the food-related antisaccade task will be analysed. Further, we will assess different components of impulsivity by self-report measures via the *UPPS Impulsive Behavior Scale* [31] and the *Process analyses of impulsive behaviours* [49]. Following measures will be assessed:

- Error rate and latency of antisaccades during the food-related antisaccade task at T0, T1 to T6, and T7.
- Quantification of impulsivity (negative urgency, premeditation, perseverance, sensation seeking) at T0, T7 and T8
- Frequency of impulsive behaviours at T0, T4, T7 and T8

##### Motivation

Motivation will be assessed by using the *University of Rhode Island Change Assessment* (URICA-S) [8]. Following measures will be assessed:

- Quantification of Precontemplation, contemplation, change for action and maintenance at T0, T7 and T8

##### Self-esteem

Self-esteem will be assessed by the *Rosenberg Self-Esteem Scale* (RSE) [46]. Following measures will be assessed:

- Quantification of global self-esteem at T0, T7 and T8

##### Self-efficacy

General Self-efficacy will be assessed by the *Internal–External locus of control* (IE-4) [33], food-related self-efficacy will be assessed by a novel self-report questionnaire by Krey (2019). Following measures will be assessed:

- Quantification of internal and external locus of control at T0, T7 and T8
- Quantification of food-related self-efficacy concerning certain foods, specific situations and general food-related self-efficacy at T0, T7 and T8

##### Treatment expectation and evaluation

Treatment expectation will be assessed by a self-developed self-report evaluation sheet which focusses on app-based training programmes and their expected effectiveness. Following measures will be assessed:

- Quantification of factors concerning subjective need, motivation for training uptake and overall satisfaction at T0 (treatment expectancy) and T7 (treatment evaluation)
- Feasibility and acceptability of the treatment will be determined by the percentage of included patients from the eligible patients at T0 and the drop-out rate at T9

##### Valence ratings

Rating of stimulus valence of the food pictures is assessed based on visual analogue scales (−5 to 5). Next to the food images being used in the inhibitory control training which can be subsumized as high-caloric food, stimulus valence of low-caloric food pictures are assessed as well [9]. Following measures will be assessed:

- Valence („very unpleasant “ to „very pleasant “), appetite („very unappetizing “ to „very appetizing “), wanting („not at all “ to „very much “) and liking („not at all “ to „very much “) at T0 and T7

### Sample size

We base our intended sample size on our randomized-controlled clinical pilot trial [19], where 20 patients per group were sufficient to detect significant effects with a moderate effect size between two groups (treatment vs. active comparator; d ≈ 0.7). With an expected dropout rate of 5% according to Giel et al. [19], we decided to allocate 63 patients in our current trial (n = 21 per study arm).

### Randomization

Randomization will be performed by a staff member of the Department of Psychosomatics and Psychotherapy after finishing the diagnostic assessment at T0. People get randomized in a 1:1:1 ratio to a) inhibitory control training with feedback and post-assessment, b) inhibitory control training without feedback and post-assessment, c) waitlist-control and post-assessment. All arms undergo the four- and twelve-week follow-up.

### Blinding

The raters assessing psychopathology are blind regarding the treatment condition. Patients who are randomised to the a) inhibitory control training with feedback and b) the inhibitory control training without feedback will be blinded towards treatment conditions, i.e. prior to the study they will be informed that only one element of the training differs between the two conditions, and they will be informed about this difference in more detail after termination of the study. Raters conduct the appointment T0, T8 and T9 which are separate from the intervention (T1-T6)/waitlist-control and post-assessment (T7) appointments, thus ensuring blinding to the treatment condition.

### Data management

All data is assessed pseudonymized. Data will predominantly be entered manually by either the study personnel or patients themselves for self-report measures. Data from paper-based source data will later be transferred to an electronic study database and fidelity and plausibility to the source data will be checked at random by the study staff. All trial data will be stored in line with the European General Data Protection Regulation 2018.

### Statistical methods

The primary analysis will use the number of binge eating episodes during the last four weeks in a linear mixed model with baseline adjustment at T8 and T9. In case of non-normally distributed data we want to use generalized linear mixed models as this statistical approach accounts for non-normally distributed data better. To test for noninferiority of the inhibitory control training without feedback compared to the inhibitory control training with feedback, we perform one tailed-tests with an alpha-level of 0.025. Further, we compare if the confidence interval of the inhibitory control training without feedback includes the effect of the inhibitory control training with feedback which resembles the non-inferiority margin [3, 37, 53]. For comparison of inhibitory control training with feedback against the waitlist control and for the comparison of inhibitory control training without feedback against the waitlist control we conduct two-sided tests with an alpha-level of 0.05.

In secondary analyses, a mixed model approach will be used to analyze the secondary outcomes also with baseline adjustment. Effect sizes will be analyzed and reported for primary and secondary outcomes. For binary outcomes, odds ratios and for quantitative outcomes, standardized differences will be reported. Primary parameters will be time vs. treatment interactions at several time points after baseline.

Further, for the regression analyses, a mixed model approach will be used as well. We want to investigate the impact of sociodemographic, comorbid psychopathology (SKID-V, BDI-II), self-efficacy (URICA-S, IE-4, food-related self-efficacy), self-esteem (RSE), treatment expectation (self-developed self-report questionnaire) as predictors on the primary outcome.

Last, for potential mechanisms underlying the inhibitory control training, we will conduct mixed models on the valence ratings of the different food stimuli and its relation to the primary outcome.

There will be no interim analysis. The primary analysis will be done for the intent to treat population, which is defined by including all patients who attended the baseline assessment. Multiple imputation will be used for subjects dropping out according to the method of Rubin. The imputation model will use 500 replications and will include age, gender and baseline assessment. Furthermore, available values from preceding visits will be included. Analyses will be performed using SPSS for Windows (Version 28.0.0.0) and R (Version 4.2.2.) using the packages *lme4* [6], *lmerTest* [34] and *emmeans* (Lenth, 2019).

### Ethical aspects

Ethical approval for conducting the trials has been obtained by the ethics committee of the Medical Faculty and the University Hospital Tübingen (Reference Number 679/2023BO2). All trial patients provide written informed consent prior to inclusion into the study. Patients can withdraw from the trial an any point without any disadvantage. Patients receive monetary compensation (8€/h).

## Discussion

The MINDBinges trial aims to identify the underlying mechanisms of a food-related inhibitory control training for individuals suffering from regular binge eating, as seen in BED, BN, OSFED and UFED with regular binge eating behaviour. This trial seeks to enhance our understanding of factors predictive of effective treatment, thereby enabling the development of more targeted cognitive trainings that address specific facets of eating disorder pathology.

The primary aim of this trial is to disentangle the role of feedback and its clinical relevance within cognitive trainings. To the best of our knowledge, this is one of the first studies in the domain of eating disorders investigating the role of feedback on task performance on clinical outcomes. This study builds on findings from a previous clinical RCT, which demonstrated a significant strong reduction in binge eating in a clinical population following six sessions of the same food-related inhibitory control training [19]. As in the prior study, we employ an individualized training approach by selecting food stimuli according to personal preference. The primary outcome reflects high clinical relevance as binge eating frequency is the core pathology of the assessed eating disorders (American Psychiatric Association and Association, 2013). Importantly, a lived experience representative has been part of the study team from the beginning of the project and was involved in the conceptualization of the whole study, especially regarding the development of the research questions. The perspective of this lived experience representative on the role of feedback is summarized in Box 1. The role of feedback is hypothesized to have a valuable role in optimizing inhibitory control trainings. To assess longer-term changes, we also stick to a comparably long follow-up period, enabling conclusions on partial or full remission of study patients. Additionally, we also investigate the superiority of the treatment against a waitlist control which was absent in the previous trial. Finally, by adopting a transdiagnostic approach and using a broad range of questionnaires for an in-depth characterization of the study population which might offer chances in subgroup-typical specifics in adapting and increase specifity of cognitive trainings.

A deeper understanding of underlying mechanisms driving the effectiveness of cognitive trainings is of particular interest. Several potential pathways have been proposed, for example the *devaluation effect* of food stimuli [56], healthy food choices [30] but also eating-disorder-specific traits [10, 16]. Additionally, unspecific traits like self-efficacy, metacognitive self-regulation and self-esteem may also predict positive treatment outcomes [44]. To get a broad view on several variables that might be causal in the treatment outcome we included several variables related and unrelated to the eating disorder to allow for conclusions on effective treatment based on individual predispositions.

Nonetheless, the current trial faces several challenges: In the previous RCT, participants had a 50 percent chance of receiving real transcranial direct current stimulation (tDCS) and still received the inhibitory control training when assigned to the placebo tDCS, which likely enhanced overall acceptability. In contrast, participant engagement in the present trial may be more difficult to ensure, as only the training or a waitlist condition are provided. Moreover, the training alone may not fully address the necessity of multimodal treatment which is demanded by severity of BED and BN [25]. Further, to work out the importance of food-specific ICT, it might make sense to consider another control group instead of a waitlist group: a group receiving a sham training, e.g. ICT with neutral/non-food specific stimuli, could meet these criteria. Last, of study patients might be challenging, in particular to distinguish the effect of the two-weeks ICT intervention from longer-termed concomitant care.

If the training without feedback, i.e. without eye tracking, proves effective in reducing binge eating frequency to a clinically meaningful degree, and ideally achieves outcomes comparable to the training with feedback, this could pave the way for an economically viable digital intervention. Moreover, findings from this trial, including regression analyses, will allow us to examine how sample characteristics influence the effectiveness of inhibitory control training and assess its suitability for potential home-treatment. With the identification of fruitful elements of effective inhibitory control training, the foundation stone is laid for the development of app-based smartphone interventions, which already have a place in basic care of mental disorders [47].

With the data retrieved from the MIND BINGES trial, we contribute to the development of scientific well-founded treatment for individuals suffering from regular binge eating to achieve more sustainable abstinence from binge eating, even possibly being enhanced by the predictive power of subgroups being able to profit most from those intervention.

## Supplementary Information

Additional file1 (DOCX 13 kb)

## Data Availability

No datasets were generated or analysed during the current study.
