# Supplementary material for: Mechanisms of an inhibitory control training to reduce binge eating behaviours: study protocol of the randomized controlled proof-of-principle MIND BINGES trial
Source: J Eat Disord. 2025 Aug 7;13:170. doi: 10.1186/s40337-025-01358-z (PMC12329953; doi:10.1186/s40337-025-01358-z)
Supplement: Supplementary file 1 — Additional file1 (DOCX 13 kb) [file 40337_2025_1358_MOESM1_ESM.docx]

I have undergone treatment for Binge Eating disorder as a study participant in a prior study investigating the same training task, and I am currently part of the research group for MindBinges. I was very happy to bring my opinion on the importance of feedback to the discussion and seeing it implemented in the project.

For me, researching the role of feedback seems very important for two main reasons:

1. The feedback about my task performance was and still is very helpful to me. The given feedback motivated me very much. It pushed me to increase my performance especially when I got told how many mistakes I had made in the task.
2. In my professional job as a teacher, I experience as well how important feedback is. Moreover, feedback can be perceived differently. How feedback is perceived by a person varies for different reasons, and I’m happy that the current study addresses the question, what factors lead to a positive impact of feedback. For example, what might motivate one person might demotivate another person, depending on their life experiences.

Therefore, I look forward to retrieve insights on the importance and function of feedback with regards to a successful treatment of binge eating disorder.
